# A novel gene functional similarity calculation model by utilizing the specificity of terms and relationships in gene ontology

**DOI:** 10.1186/s12859-022-04557-6

**Published:** 2022-01-20

**Authors:** Zhen Tian, Haichuan Fang, Yangdong Ye, Zhenfeng Zhu

**Affiliations:** School of Information Engineering, Zhengzhou University, Zhengzhou, China

**Keywords:** Gene ontology, Information content, Specificity of terms and edges, Gene functional similarity

## Abstract

**Background:**

Recently, with the foundation and development of gene ontology (GO) resources, numerous works have been proposed to compute functional similarity of genes and achieved series of successes in some research fields. Focusing on the calculation of the information content (IC) of terms is the main idea of these methods, which is essential for measuring functional similarity of genes. However, most approaches have some deficiencies, especially when measuring the IC of both GO terms and their corresponding annotated term sets. To this end, measuring functional similarity of genes accurately is still challenging.

**Results:**

In this article, we proposed a novel gene functional similarity calculation method, which especially encapsulates the specificity of terms and edges (STE). The proposed method mainly contains three steps. Firstly, a novel computing model is put forward to compute the IC of terms. This model has the ability to exploit the specific structural information of GO terms. Secondly, the IC of term sets are computed by capturing the genetic structure between the terms contained in the set. Lastly, we measure the gene functional similarity according to the IC overlap ratio of the corresponding annotated genes sets. The proposed method accurately measures the IC of not only GO terms but also the annotated term sets by leveraging the specificity of edges in the GO graph.

**Conclusions:**

We conduct experiments on gene functional classification in biological pathways, gene expression datasets, and protein-protein interaction datasets. Extensive experimental results show the better performances of our proposed STE against several baseline methods.

## Background

Since Gene Ontology (GO) [1, 2] was first founded in 1998, it has been an important resource to support modern biological research. The GO knowledge base contains a controlled vocabulary of terms, which has three different orthogonal ontologies named biological process (BP), molecular function (MF), and cellular component (CC) . In each ontology, terms are employed to describe the function of genes and the relationships which have specific meanings are used to connect two terms. There are many relationships in the GO database and we only consider two of them: is_a and part_of.

GO exists in the form of a directed acyclic graph (DAG) and has two important characteristics. One is that terms with lower hierarchy generally show more specific meanings while terms with higher hierarchy have more generic meanings. Traditionally, the specific and generic meanings of terms are measured by the IC values, which could represent their specificity. The other is that the edges in different levels also have different specificity because of the terms that they connect.

The functions of a gene could be described by go terms and thus we suggest that this gene is annotated by the terms. The GO annotations (GOA) [3–5] database is specifically used to describe genes and their annotation terms. Since GO has three branches, genes can also be annotated from BP, CC, and MF aspects. Comparing the functional similarity between genes has many significant applications [6–10], such as protein interaction prediction, gene clustering, and disease gene identification.

In the past decades, various kinds of methods have already been developed for studying gene functional similarity. The most important concept in functional similarity comparison of genes domain is IC, which could measure the specificity of a GO term. So far, there are two types of IC values computing categories: corpus-based [11–17] and structured-based [18–21].

For a term *t*, its IC value calculated by corpus-based approaches shows as follow:

1 $$\begin{aligned} I C_{corpus}(t)=-\log (p(t)) \end{aligned}$$

where *p*(*t*) denotes the probability of both term *t* and its descendants appearing in the corpus. Method Resnik [11], Jiang and Conrath [12], and Lin [13] are all based on this definition. Equation 1 strongly demonstrates that the IC value of a given GO term is mainly attributed to the number of genes or proteins it annotates in the corpus. Therefore, the IC value of terms may vary according to the corpus. On the other hand, the annotation information in a corpus is updating over time, which also has an effect on the IC values of terms [22].

To overcome this drawback, researcher David Sánchez [18] put forward another IC computing model based on the GO structure. For a given GO term *t*, its IC value can be expressed as:

2 $$\begin{aligned} IC_{structure}(t)=-\log \left( {\frac{\frac{\left| leaves(t) \right| }{\left| subsumers(t) \right| } +1 }{max\_leaves+1}}\right) \end{aligned}$$

where $max\_leaves$ means the amount of leaf terms. *subsumers*(*t*) is the ancestor set of term *t*. Additionally, the terms in *leaves*(*t*) are belonged to the descendants of term *t* that are also belonged to leaves. From the equation, we can find that this model exploits the specific genetic information of term *t*. The information contains the leaves of ontology, the number of their descendants and ancestors. Later, method SORA [19] and WIS [20] make an improvement based on this model, and achieve better performances.

Based on the IC values of terms, researchers have developed numerous gene functional similarity methods, which have two categories: pair-wise strategies [11–13, 23] and group-wise strategies [19, 20, 24–28].

For pair-wise strategy methods, they measure gene functional similarity mainly utilizing two steps: the first one focusing on computing the semantic similarity between annotated terms and the second one is measuring functional similarity with respect to the semantic similarity in the first step. The best matches average rule is commonly used in the second step. For group-wise approaches, they measure the gene functional similarity from the annotation set perspective. Here we select some typical computing models for a brief review. A detailed review is beyond the scope of this paper and has already been presented by Catia Pesquita [29].

Method Resnik [11] is a pair-wise strategy approach. For two given term $t_1$ and $t_2$, the semantic similarity between them can be expressed as:

3 $$\begin{aligned} Sim_{Resnik}(t_1,t_2)=IC(LCA(t_1,t_2)) \end{aligned}$$

where $LCA(t_1, t_2)$ means the lowest common ancestor for term $t_1$ and $t_2$. Then it calculates gene functional similarity leveraging the BMA rule. The procedure for some other methods like Wang [23], Jiang and Conrath [12], and Lin [13] are similar to method Resnik.

Method simUI [26] is a group-wise method, which is proposed by Gentlman. Suppose there are two genes $G_1$ and $G_2$, the formula of this model can be expressed as:

4 $$\begin{aligned} simUI(G_1,G_2)=\frac{\left| S_{G_1}\cap S_{G_2}\right| }{\left| S_{G_1}\cup S_{G_2}\right| } \end{aligned}$$

where $S_{G_1}$ and $S_{G_2}$ represent the annotation term set for $G_1$ and $G_2$ respectively. Followed Gentlman, some other models such as SimGIC [27], SORA [19] and WIS [20] are also proposed. All of these approaches pay much attention to compute the IC of annotated term sets accurately and effectively. For example, simGIC sums up the IC value of each term, while SORA puts forward the concept of inherited semantics to avoid computing the overlap IC of terms in annotation sets. Method WIS first assigns a weighted value to the relationships of GO structure and then designs a rule to compute the inherited IC values of GO terms.

Based on the idea of vector representation, some other approaches [24, 25, 30] are proposed. These methods employ the one-hot coding to deal with the annotation terms. Terms in the annotation term set will be represented in a vector of which dimension indicates the total amount of GO terms. Each dimension denoted by a binary digit. Suppose there are two genes $G_1$ and $G_2$, their annotation term vectors are $v_1$ and $v_2$, the functional similarity between $G_1$ and $G_2$ based on basic vector space model (VSM) can be expressed as:

5 $$\begin{aligned} Sim_{VSM}(G_1,G_2)=\frac{\upsilon _1\cdot \upsilon _2}{\left| \upsilon _1 \right| \left| \upsilon _2 \right| } \end{aligned}$$

For group-wise approaches, they do not make the best use of the GO structure, which may cause the calculation of IC not accurately. For example, method GIC does not take the number of ancestors of terms into consideration on the IC values calculation. VSM neglects the relationship between GO terms.

To overcome the drawbacks, we put forward a novel gene functional similarity calculation method, which especially encapsulates the Specificity of Terms and Edges (STE). STE mainly has two models: the first one calculates the IC value of terms and another one is designed for computing the weighted value of edges. Their detailed description will be shown in “Methods” section.

## Results

In this section, the experimental results on various datasets are presented. Before that, we first introduce the experimental data.

### Datasets

The GO data is downloaded from the online resource website. In this version, the term number of BP, CC, and MF are 29,380, 4,181, and 11,113 respectively. Besides, the Gene Ontology annotation for H. sapiens and Saccharomyces cerevisiae data are also downloaded from the gene ontology resource website. In this study, we divide annotation types into two categories: IEA+, and IEA-, which means that the annotation term sets of genes contain the Inferred electronic-assigned (IEA) terms or not. Moreover, six annotation combinations are presented as MF_IEA+, MF_IEA-, CC_IEA+, CC_IEA-,BP_IEA+,BP_IEA-.

It is an important and popular validation strategy for gene functional similarity methods to classify the genes based on molecular function. In this study, we employ the yeast pathway data in Saccharomyces genome database (SGD) to make an analysis for the functional classification of genes based on the gene functional similarity calculation results.

For protein-protein interaction experiments, we download the data from the previous approaches [20, 23]. Besides, we remove the obsoleted data and rebuild a new experimental dataset. Negative PPIs for human and yeast are randomly generated based on the annotation of genes on three ontologies. What’s more, the number of negative PPIs and positive PPIs are the same.

In the end, gene expression data of Saccharomyces cerevisiae is from Jain and Davis [31]. In this dataset, there are a total of 11,966 pairs of cerevisiae gene when we remove some obsoleted data. In the end, there are a total of 4,211, 3,888, and 3,867 gene pairs for CC, BP, and MF aspects respectively.

### The analysis for the distribution of IC

**Fig. 1:**
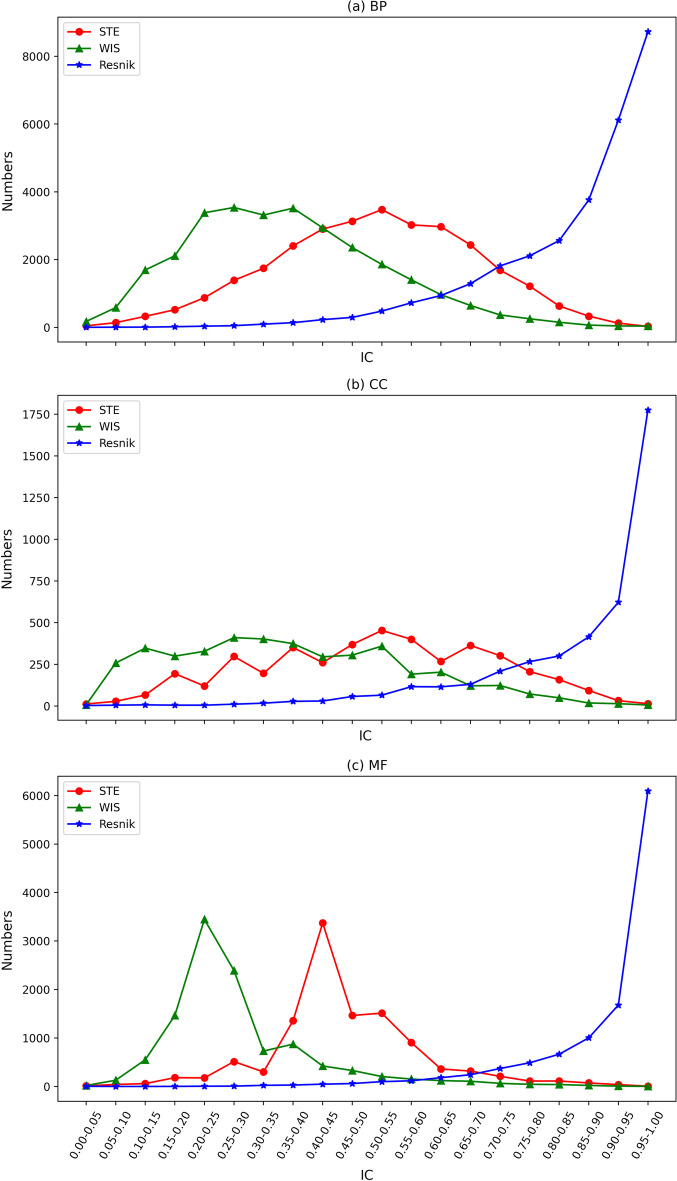
The amount distribution of terms based on IC with respect to ontologies **a** BP, **b** CC, and **c** MF. X-axis and Y-axis indicate the the scope of IC value and the amount of terms respectively

**Fig. 2:**
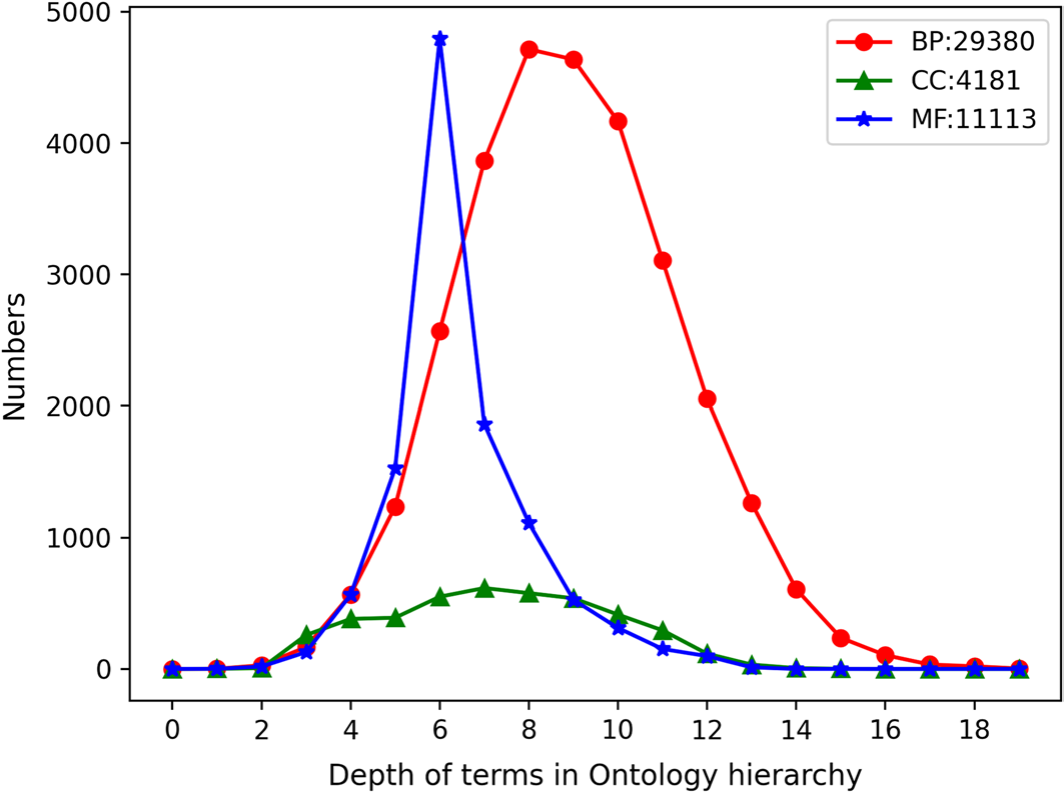
The amount distribution of terms based on depth with respect to BP, CC and MF ontologies

Measuring the IC of GO terms reasonably is the foundation for accurately calculating the gene functional similarity. Therefore, we firstly investigate the distribution of the IC of terms on three sub-ontologies with three methods which are Resnik, WIS, STE. The detailed results are shown in Fig. 1. According to Eq. 6, terms with higher levels tend to have smaller IC values, while terms with lower levels have bigger IC values. To prove this point of view, we investigate the relationships between the number of terms and their depth. The results are shown in Fig. 2. The GO terms with the middle level are 89 percent of the total in the three ontologies, which demonstrates that the IC values of most terms should be medium.

Lastly, we analyze the results of method Resnik, WIS, and STE in detail. For method Resnik, there is more than 85 percent of IC of terms are larger than 0.9. In other words, this model does not distinguish the difference of terms in the GO graph. Method WIS makes a big improvement compared with Resnik. However, many small IC values are presented on the curve of WIS. The results of the proposed model STE are highly consistent with the Eq. 6, which meets the human perspectives. Overall, STE is the best model in these three methods in measuring the IC values of terms.

### Gene functional classification in biological pathways

**Fig. 3:**
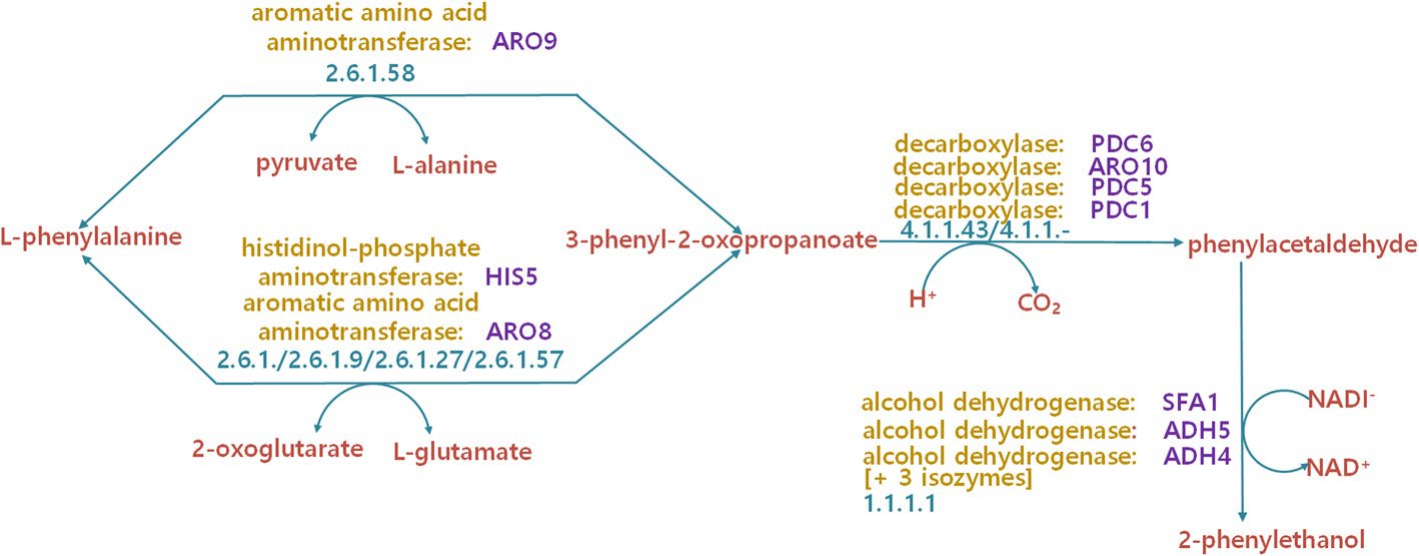
The detailed information of phenylalanine degradation pathway

Compare gene functional similarity calculation methods with meaningful pathways is effective to a large extent. If the results of a gene functional similarity method are consistent with the fact that demonstrated in the biological pathways, this method will be an effective one. Meanwhile, there are more than 80 biological pathways in the selected dataset, and we choose one pathway named ‘phenylalanine degradation’ with ten different genes and eight various EC numbers to validate the performances of methods to be compared. The selected pathway is shown in Fig. 3. At the same time, we compute the functional similarities of the 10 genes with respect to MF ontology with STE and three baseline methods Resnik, Wang, and VSM.

**Fig. 4:**
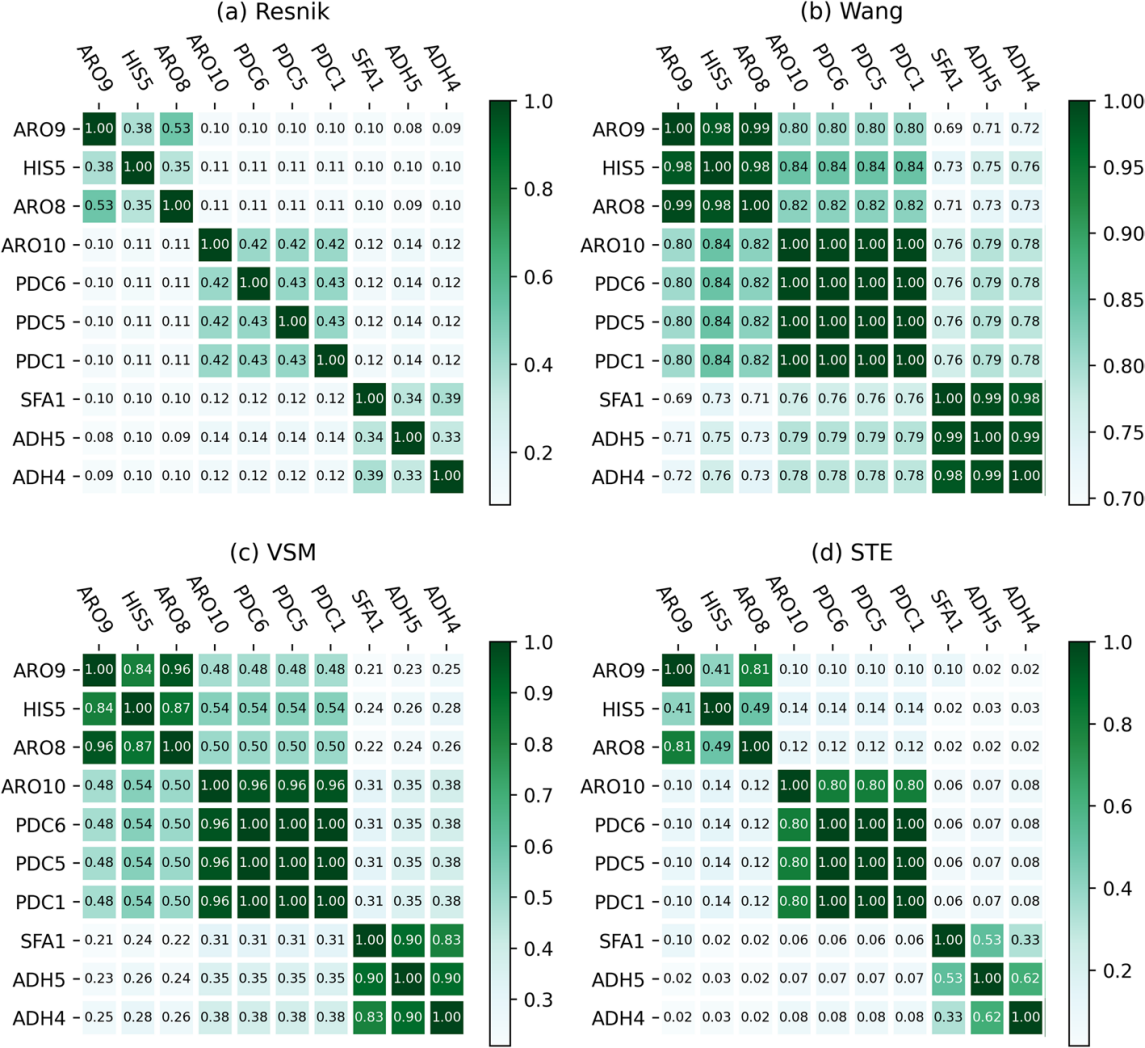
Gene function classification results with respect to MF ontology. **a** Resnik, **b** Wang, **c** VSM and **d** STE

It is generally believed that genes with similar EC numbers will have a higher functional similarity. The results are demonstrated in Fig. 4. For method Resnik, there is one pair of genes of which functional similarity is higher than 0.5 and the similarity of other genes pairs are small. Taking gene ‘PDC1’ and ‘PDC5 ’ as an example, the EC number of these two genes are the same, and their similarity value is only 0.43. For method Wang, the functional similarities of gene pairs are not very distinguishable. For example, gene ‘PDC6’ has a higher similarity with gene ‘ADH5’ and ‘ADH4’ than gene ‘SFA1’. This is not inconsistent with the EC number knowledge. For method VSM, the EIC number of gene ‘H1S5’ and ‘PDC1’ is quite different, but the functional similarity of them has a higher similarity, which is unreasonable.

In the end, for STE, the functional similarities of gene pairs are consistent with the class of the EC number. Moreover, it can also distinguish different the ‘distance’ of gene pairs from the functional similarity. Overall, STE is the best of the four compared methods.

### Results on PPIs

It is another critical evaluation criterion to score the functional similarity calculation methods utilizing protein interactions. In this sub-experiment, according to the selected PPIs in the dataset, we calculate their functional similarity. Then the performance of functional calculation methods is deeply compared based on the receiver operating characteristic (ROC) and the area under the curve (AUC) metric.

The functional similarity values of PPI pairs for S. cerevisiae and H. sapiens are measured using all seven methods. Tables 1 and 2 present their corresponding AUC values respectively. Specifically, on S. cerevisiae dataset, method simGIC runs first on CC_IEA- and MF_IEA+. STE achieves the best performance on four sub-datasets, which are CC_IEA+, BP_IEA+, MF_IEA-, and BP_IEA-. The performances of the other four methods are inferior to these two methods on the whole. On H. sapiens dataset, similar to the results on S. cerevisiae, method STE get the rank first on three sub-ontologies: BP_IEA+, BP_IEA- and MF_IEA-. Besides, simGIC achieves a relatively good performance, since it also got first results on MF_IEA+, CC_IEA+ and CC_IEA-. However, there is only a small gap between STE and simGIC on CC_IEA- that the score of simGIC is 0.0028 higher than that of STE. Therefore, method STE is superior to method simGIC and the other five methods on PPI experiments. It is worth noting that group-wise methods show better performance than pairwise methods on PPI experiment.

**Table 1:** AUC values in S. cerevisiae datasets with respect to ontology BP, CC and MF (IEA+ and IEA-)

Methods	BP_IEA+	CC_IEA+	MF_IEA+	BP_IEA-	CC_IEA-	MF_IEA-
STE	**0.8234**	**0.8317**	0.7441	**0.8724**	0.8343	**0.7460**
simGIC	0.8198	0.8223	**0.7497**	0.8647	**0.8392**	0.7023
Resnik	0.7888	0.8211	0.6987	0.7949	0.8043	0.6182
WIS	0.8184	0.8249	0.7371	0.8643	0.8122	0.7259
simUI	0.8095	0.8213	0.7253	0.8447	0.8004	0.7098
VSM	0.8115	0.8246	0.7294	0.8477	0.8033	0.7088
Wang	0.7932	0.8028	0.7110	0.8262	0.7948	0.6905

The best results are in bold

**Table 2:** AUC values in H. sapiens datasets with respect to ontology BP, CC and MF (IEA+ and IEA-)

Methods	BP_IEA+	CC_IEA+	MF_IEA+	BP_IEA-	CC_IEA-	MF_IEA-
STE	**0.8624**	0.7504	0.7228	**0.7940**	0.6839	**0.6907**
simGIC	0.8381	**0.7614**	**0.7597**	0.7839	**0.6867**	0.6730
Resnik	0.6696	0.6714	0.7033	0.7264	0.6638	0.6662
WIS	0.8049	0.6734	0.6637	0.7718	0.6604	0.6835
simUI	0.7921	0.6484	0.6208	0.7734	0.6586	0.6836
VSM	0.7896	0.6564	0.6297	0.7825	0.6675	0.6732
Wang	0.7334	0.6260	0.5824	0.7404	0.6466	0.6474

The best results are in bold

### Results of gene expression experiment

In this experiment, we randomly selected 3500 gene pairs on the three ontologies. At the same time, the functional similarity with IEA+ and IEA- on different ontologies of these gene pairs are computed using our proposed STE and six baselines (WIS, VSM, Resnik, Wang, simUI, and simGIC). Based on the obtained gene functional similarity values and gene expression values, the pearson’s correlation coefficients between them are calculated. The results for these seven methods are listed in Table 3.

**Table 3:** Pearson’s correlation coefficient with gene expression dataset with respect to ontology BP, CC and MF (IEA+ and IEA-)

Methods	BP_IEA+	CC_IEA+	MF_IEA+	BP_IEA-	CC_IEA-	MF_IEA-
STE	0.4048	0.4197	0.2411	0.4403	0.5412	**0.1998**
simGIC	**0.4053**	0.4212	**0.2546**	**0.4418**	**0.5540**	0.1972
Resnik	0.3135	**0.4405**	0.2219	0.3818	0.5286	0.1439
WIS	0.3993	0.4125	0.2457	0.4318	0.5162	0.1980
simUI	0.3799	0.4003	0.2241	0.4252	0.5151	0.1889
VSM	0.3416	0.3621	0.1941	0.4024	0.4999	0.1806
Wang	0.2160	0.2292	0.0695	0.3141	0.4046	0.0563

The best results are in bold

On the whole, the correlation coefficients on CC, BP, and MF have a different distribution that CC ontology has the highest values, followed by BP and MF. On the method aspect, method GIC performs best on four sets of experiments, which are BP_IEA+, MF_IEA+, CC_IEA-, and BP_IEA- respectively. Meanwhile, method Resnik get, the highest score on CC_IEA+. The proposed method STE only runs first on MF_IEA-, which is less unsatisfactory. In this experiment, the performance of method simGIC is best, followed by method STE and Resnik. On the whole, the performance of group-wise approaches is better than that of the pairwise methods.

## Discussion

In the current study, we propose a novel computational model for calculating the IC of a term in the GO graph. As far as we know, there are two categories of methods for computing the IC of GO terms, which are corpus-based and structural-based. Corpus-based methods such as Lin [13], Jiang and Conrath [12], and Resnik [11], measure the IC of a term by calculating the frequency that the term appears in a specific corpus. However, owing to the diversity and variability of corpora, corpus-based methods may obtain inaccurate IC of terms. Structural-based methods incorporate the structural information of term into its IC, which can effectively capture the information of the GO graph. For example, Sánchez [18] uses the ancestors of terms and the leaf terms as the information to measure IC. Subsequently, SORA [19] propose to add the depth information of terms into the measurement of IC. Following SORA, WIS [20] employ the depth, ancestors, descendants simultaneously to enrich the information contained by terms. Nevertheless, Sánchez may lose some useful structural information such as depth compared with SORA and WIS. WIS improves SORA by introducing the depth of descendants and the number of ancestors and . These works show the IC of a term has a strong correlation with its depth in the GO graph.

Inspired by the above observations, we encapsulate the depth of both the given term and its ancestors to compute its IC. Additionally, we also exploit the number of descendants and all GO terms to enrich the information contained by terms. From Fig. 2, it can be found that a large proportion of terms located in the middle hierarchy of the GO graph. Further, Fig. 1 demonstrates the IC of terms calculated by our proposed method are concentrated in the middle range, whether in BP, CC, or MF, which fits well with the distribution in Fig. 2. To sum up, our proposed method for calculating the IC of terms is more effective against the early proposed methods.

## Conclusion

In the current study, we proposed a novel computational model called STE to measure gene functional similarity. This method could make the best use of the GO structure to calculate the IC values of GO terms accurately by assigning a reasonable weighted value to the relationships of the GO structure. Especially, the depth and the genetic structure of GO terms are all merged into the IC value calculation model. Therefore, the IC values of terms are ranging from 0 to 1 and most of them are between 0.3 and 0.7. Besides, based on the values of edges, we have the ability to accurately estimate the IC values of annotation term sets with the concept of the inherited IC value concept. This is critical to the functional similarity calculation methods. Consequently, experimental results on various datasets demonstrated that STE is superior to the other six competitive methods in measuring functional similarity of genes.

## Methods

### Measuring the IC value of a term

A GO term with a lower level will describe a more specific function and vice versa. The IC of a term will be employed as a metric to measure how specific the term is. Therefore, terms with lower hierarchy will show higher values than those with higher hierarchy. Aside from this, terms with lower hierarchy always tend to have more ancestors and fewer descendants. Therefore, for a give GO term *t*, a novel computational model for calculating its IC value is developed as follows:

6 $$\begin{aligned} \begin{aligned} IC(t)=log(depth(t))&*\left( log\left( \sum \limits _{t_i\in Ance(t)}depth(t_i)\right) +1 \right) \\&*\left( 1-\frac{log(\left| Desc(t)\right| )}{log(N+1)} \right) \end{aligned} \end{aligned}$$

where *Ance*(*t*) and *Desc*(*t*) denote the ancestor set and descendant set of term *t*, *depth*(*t*) and *N* are the max depth of term *t* and the total amount of GO terms.

### The weighted value of an edge

As we know, there are many edges at different levels that linking the terms in the GO graph. To show the specificity of the edge, we assign a value ranging from 0 to 1 to each edge in the GO graph. The model for calculating the weighted value of an edge between term $t_i$ and $t_j$ can be expressed as:

7 $$\begin{aligned} \omega _{ij}=\frac{\sum \limits _{t_m\in Desc(t_j)}IC(t_m)}{\sum \limits _{t_n\in Desc(t_i)}IC(t_n)} \end{aligned}$$

where $t_j$ is the direct descendant node of $t_i$, *Desc*(*t*) contains the descendants of term *t*.

### Measuring the own IC of a term

Based on the true path rule in the gene annotation area, if one gene is annotated by a GO term, all the ancestors of this term will annotate the gene. Therefore, terms in the annotation term set have an inheritance relationship. The IC of term $t_j$ can be seen as two parts: one is the inherited IC denoted as $IC_{inherited}(t_j)$ from its parent terms and the other is its own IC denoted as the $IC_{own}(t_j)$, which can be calculated as follows:

8 $$\begin{aligned} IC_{inherited}(t_j)=\sum _{t_i\in Parent(t_j)}\omega _{ij}*IC(t_i) \end{aligned}$$

where $Parent(t_j)$ includes all the direct ancestors of term $t_j$. The weighted value $\omega _{ij}$ is calculated using Eq. 7.

9 $$\begin{aligned} \begin{aligned} IC_{own}(t_j)&=IC(t_j)-IC_{inherited}(t_j) \end{aligned} \end{aligned}$$

### Measuring the IC value of a term set

To avoid calculating the overlap semantics in a term set, we utilize Eq. 9 to sum up the IC of a term. Suppose *T* is a term set, the IC value of *T* defined as follows:

10 $$\begin{aligned} IC(T)=\sum \limits _{t\in T}IC_{own}(t) \end{aligned}$$

### An example of calculating the IC of a term set

**Fig. 5:**
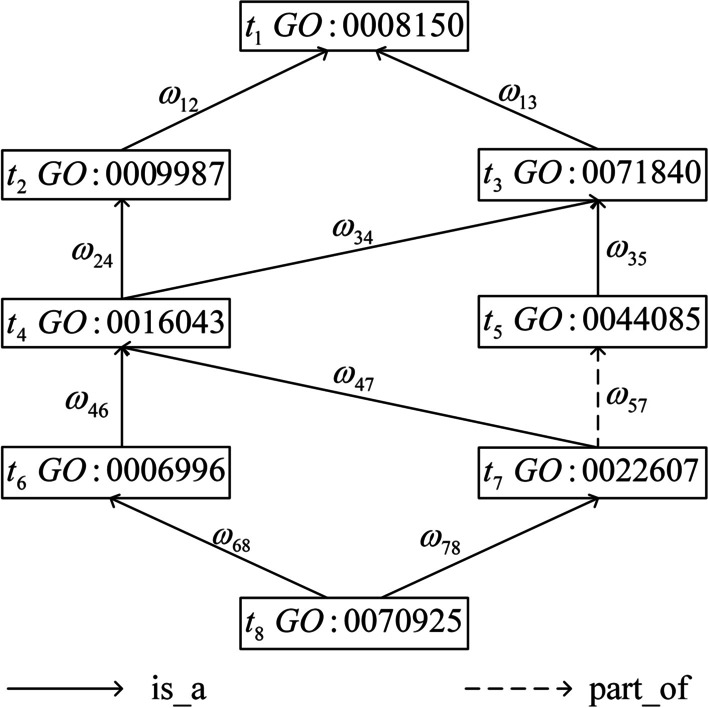
DAG for GO term organelle assembly:0070925

From Fig. 5, eight GO terms are contained in a term set. The IC of each term and the weight of every edge are listed in Tables 4 and 5 respectively. Suppose set *S* contains the eight terms and the computational process using our method to calculate the IC of term *S* is presented in Table 6.

**Table 4:** The IC values of corresponding terms in Fig. 5

Term	$t_1$	$t_2$	$t_3$	$t_4$	$t_5$	$t_6$	$t_7$	$t_8$
IC	0.0	0.01	0.02	0.04	0.05	0.07	0.09	0.18

**Table 5:** The weight values of corresponding edges in Fig. 5

Edge	$\omega _{12}$	$\omega _{13}$	$\omega _{24}$	$\omega _{34}$	$\omega _{35}$	$\omega _{46}$	$\omega _{47}$	$\omega _{57}$	$\omega _{68}$	$\omega _{78}$
Weight	0.85	0.98	0.97	0.84	0.71	0.65	0.71	0.72	0.67	0.84

**Table 6:** The computational process for measuring the IC of term set S

Step	Term	*IC*	$IC_{own}$	*IC*(*S*)
1	$t_1$	0.0	0	0.000
2	$t_2$	0.01	$IC_{own}(t_2)=IC(t_2)-IC(t_1)*\omega _{12}=0.01$	0.010
3	$t_3$	0.02	$IC_{own}(t_3)=IC(t_3)-IC(t_1)*\omega _{13}=0.02$	0.030
4	$t_4$	0.04	$IC_{own}(t_4)=IC(t_4)-IC(t_2)*\omega _{24}-IC(t_3)*\omega _{34}=0.014$	0.044
5	$t_5$	0.05	$IC_{own}(t_5)=IC(t_5)-IC(t_3)*\omega _{35}=0.036$	0.080
6	$t_6$	0.07	$IC_{own}(t_6)=IC(t_6)-IC(t_4)*\omega _{46}=0.044$	0.124
7	$t_7$	0.09	$IC_{own}(t_7)=IC(t_7)-IC(t_4)*\omega _{47}-IC(t_5)*\omega _{57}=0.026$	0.150
8	$t_8$	0.18	$IC_{own}(t_8)=IC(t_8)-IC(t_6)*\omega _{68}-IC(t_7)*\omega _{78}=0.058$	0.208

In the first step, we initialize the *IC*(*S*) to be 0. It’s obvious that term $t_1$ is the root of the term set and $IC_{own}(t_1)$ is 0. Hence,the result after the first step is *IC*(*S*) plus $IC_{own}(t_1)$ and is equal to 0.

In the second step, according to the Eq. 9, we calculate $IC_{own}(t_2)$ and its result is 0.01. Therefore, the result of *IC*(*S*) after the second step is *IC*(*S*) calculated by the last step plus $IC_{own}(t_2)$ and is equal to 0.01.

After iteration is finished, we have calculated all $IC_{own}$ of terms in the DAG and get the final result of *IC*(*S*). It is worth noting that using the proposed method to calculate the IC of term sets is very efficient and Algorithm 1 describes the computational process of the IC of o term set by the proposed model.

### Measuring the gene functional similarity

Suppose there are two genes $G_1$ and $G_2$, their annotation term sets are $T_{G_1}$ and $T_{G_2}$ respectively. The functional similarity between them is expressed as:

11 $$\begin{aligned} simSTE(G_1,G_2)=\frac{IC(T_{G_1}\cap T_{G_2})}{IC(T_{G_1}\cup T_{G_2})} \end{aligned}$$

where $\cap$ denotes the intersection while $\cup$ represents the union of the two sets respectively.

## Algorithms

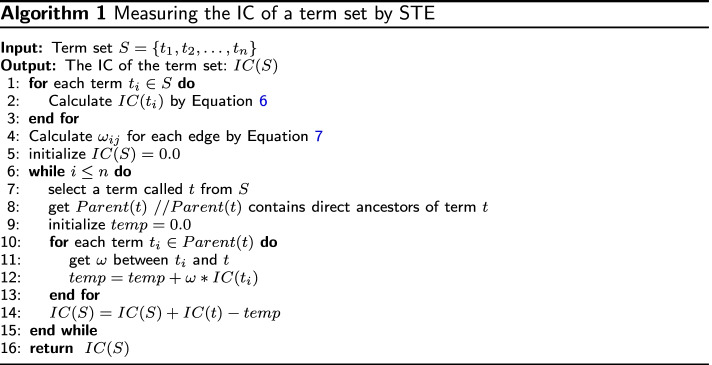



## Data Availability

The public datasets used in this study are described in the results section of this paper. The experimental data are available at: https://github.com/hc-fang/STE.

## Abbreviations

GO: Gene ontology
GOA: Gene ontology annotation
LCA: lowest common ancestor
IC: Information content
STE: Specificity of terms and edges
BP: Biological process
MF: Molecular function
CC: Cellular component
DAG: Directed acyclic graph
PPIs: Protein-protein interactions
EC number: Enzyme class number
IEA: Inferred electronic-assigned terms
MF_IEA+: Molecular function with IEA
MF_IEA-: Molecular function without IEA
CC_IEA+: Cellular component with IEA
CC_IEA-: Cellular component without IEA
BP_IEA+: Biological process with IEA
BP_IEA-: Biological process without IEA.
